# Mitochondrial function and reactive oxygen species dynamics in Italian Mediterranean buffalo semen following cryopreservation and post-thaw incubation

**DOI:** 10.3389/fvets.2025.1733446

**Published:** 2025-12-18

**Authors:** Giada Loddo, Maria Elena Gelain, Gianfranco Gabai, Asia D'Andrea, Elisabetta Montanari, Chiara Milani, Elisa Giaretta

**Affiliations:** 1 Department of Comparative Biomedicine and Food Science, University of Padova, Legnaro, Italy; 2 Intermizoo S.p.A, Caorle, Italy; 3 Department of Animal Medicine, Production and Health (MAPS), University of Padova, Legnaro, Italy

**Keywords:** Italian Mediterranean buffalo bulls, flow-cytometry, fresh semen, thawed semen, ROS production, mitochondrial function

## Abstract

**Introduction:**

The current understanding of physiological parameters and redox balance in buffalo bull semen is limited and derived from various breeds. Moreover, the effects of cryopreservation in various buffalo breeds remain unclear.

**Methods:**

This study aimed to investigate the relationships between physiological parameters and compare fresh (F) and frozen-thawed (T) semen in Italian Mediterranean buffalos (IMB; 7, bulls). Buffalo ejaculates were collected using an artificial vagina and cryopreserved using a standard protocol. Both F and T were analyzed by CASA and flow cytometry Semen parameters assessed included motility, viability (using PI or SG counterstains for each assay), acrosome integrity (PSA), mitochondrial membrane potential (JC1), mitochondrial integrity (MT), intracytoplasmic (DHE) and mitochondrial (MX) superoxide production, and other intracellular reactive oxygen species (CR). The T samples were assessed immediately after thawing (T0) and following 3 h incubation at 37°C (T3).

**Results:**

Results showed significant correlations (*p* < 0.05) between total motility (TM) and progressive motility (PM) with mitochondrial membrane potential (MMP) and mitochondrial integrity (MI). The TM, PM, MI, and MMP positively correlated (*p* < 0.05) with total reactive oxygen species (ROS) production and negatively with superoxide production. Cryopreservation significantly decreased TM from 92.1 ± 5.66% to 72.61 ± 18.62% (*p* < 0.05), H_2_O_2_ production from 23.02 ± 7.42% to 11.49 ± 8.85% (*p* < 0.05), and MMP from 83.29 ± 11.20 to 56.98 ± 15.87% (*p* < 0.05). After 3 h incubation, T semen exhibited increased mitochondrial damage and superoxide production, with decreased total ROS production.

**Discussion:**

In conclusion, cryopreservation and subsequent incubation significantly affect mitochondrial functions, which strongly correlate with sperm motility. A deeper understanding of sperm energy metabolism and its relationship with redox regulation could allow for the optimization of current assisted reproductive technologies (ART), as these factors play a crucial role in sperm viability, motility, and fertilization capacity, which are critical for optimizing outcomes in ART procedures.

## 1 Introduction

The anatomical and physiological peculiarities of the Italian Mediterranean Buffalo (IMB), a breed primarily selected for the PDO-certified “Mozzarella di Bufala Campana”, significantly affect its reproductive efficiency and the management strategies required (1–6).

Despite the economic importance of this breed, there are significant gaps in our understanding of IMB semen physiology, particularly regarding functional parameters such as energy metabolism and cellular redox homeostasis in fresh semen. The increased use of assisted reproduction technology has led to greater demand for selected breeders and available semen for artificial insemination (AI). However, studies on the functional state of IMB semen and its resistance to various storage techniques remain limited (7).

It is well known that sperm metabolism can vary significantly across species, potentially leading to different patterns of energy production and utilization that influence motility and other functional parameters (8, 9). Moreover, the impact of oxidative stress on fresh and frozen semen determines varying levels of cryotolerance, which differ not only among species but also among individuals within the same species (10).

Recent advancements in flow cytometry and computer-assisted sperm analysis (CASA) have enabled more comprehensive assessments of physiological characteristic across various species (11, 12). These techniques allow for the characterization of spermatozoa based on various biomarkers of functional states such as the intracellular oxidative status, membrane damage and mitochondrial functionality.

The freezing-thawing process is known to cause a significant reduction in sperm motility across species, with buffalo spermatozoa being particularly susceptible to cryoinjury, as reported for various buffalo breeds (13–18). Specific features of buffalo semen, such as highly condensed chromatin, low or absent mechanisms of DNA repair, high concentration of polyunsaturated fatty acids in sperm membranes, and limited cytoplasm containing antioxidant enzymes, determine a higher exposure to the oxidative stress induced by cryopreservation than more resistant species such as bull (19–23).

In IMB, it has been observed that approximately 50% of cryopreserved spermatozoa remain motile and maintain high mitochondrial membrane potential (MMP; 7, 18), suggesting a crucial role of mitochondrial activity in buffalo sperm motility.

This observation is further supported by studies on various Indian buffalo breeds, which found that the decrease in sperm motility in frozen semen can be triggered by a reduction in energy availability. This reduction is caused by either a decrease in mitochondrial membrane potential (MMP) or damage to the axonemal and membrane elements (24).

However, the scarcity of studies focusing on the physiological aspects of IMB semen highlights the need for further research in this field. Species-specific mechanisms involve both energy metabolism and cellular oxidative stress management, underscoring the importance of a more comprehensive understanding of the relationship between mitochondrial function, oxidative status, and sperm motility in IMB. Moreover, understanding the physiological adaptations to cryopreservation in IMB may be crucial for enhancing semen preservation techniques and ultimately improving the breed's reproductive efficiency.

Given these observations and the gaps in current knowledge, we hypothesize that (1) mitochondrial and oxidative status significantly influences kinematic parameters in IMB semen. (2) cryopreservation and subsequent incubation alter ROS balance beyond the buffering capacity of sperm antioxidants, impairing semen quality. This study, aims to use advanced flow-cytometric and CASA assessment to: (i) explore the correlation between oxidative status, mitochondrial functionality, and kinematic parameters in IMB semen and (ii) analyze the differences in these traits between fresh (F) and frozen-thawed (T) semen, with assessments conducted immediately after thawing (T0) and following a 3 h incubation period (T3) at 37°C.

## 2 Materials and methods

### 2.1 Study design

For the purpose of the study, two set of samples were established: fresh semen (F), and frozen-thawed samples, which were analyzed immediately after thawing (T0) and after 3 h (T3) of incubation, as tested in other previous works on buffalo semen (7, 25–28). Each experimental set was evaluated for sperm kinematic parameters and flow cytometry-based markers, including cytoplasmic and acrosomal membrane integrity, mitochondrial integrity and functionality, and intracellular reactive oxygen species (ROS) production.

### 2.2 Semen collection and preparation

Semen samples were obtained from seven Italian Mediterranean buffalo bull aged from 28 to 50 months and stabulated at the Intermizoo A.I. station in the Vallevecchia farm in Brussa, Caorle (Venice, Italy). For the analysis, at least two ejaculates per bull were collected each week from early March 2024 to mild-April 2024 and at the end of October 2024. During the semen collection period, each bull received a total mixed ration (TMR) composed of ground sorghum silage (17.5 kg as-fed), alfalfa hay (1.75 kg as-fed), straw (5.05 kg as-fed) and a protein supplement (0.84 kg as-fed) in the morning. In the afternoon, only long straw (2.0 kg as-fed) was provided. TMRs were prepared daily for all pens and offered *ad libitum*. Semen was collected using an artificial vagina and diluted to a concentration of 80 × 106 sperm/mL with a pre-warmed (37°C) extender consisting of distilled water, Tris-HCl, citric acid monohydrate, fructose, glucose monohydrate, 6.2% glycerol, egg yolk, and antibiotics. A 2 mL aliquot of fresh semen was stored at 5°C and transported in two hours to the Laboratory of the Department of Comparative Biomedicine and Food Science (BCA) at the University of Padova in Legnaro, Padova (Italy). The aliquot was then centrifugated at 800 g x 3 min twice and resuspended in 1 mL of Tyrode's medium to obtain a concentration of 80 x 10^6^ sperm/mL. After the preparation of fresh aliquots, the remaining sample was immediately cryopreserved, as protocol already published (29). Semen was diluted with the extender (25 × 10^6^ sperm/mL) and packed into 0.5 mL labeled plastic straws. This procedure was performed at 4°C. Afterwards, the straws were transferred to a programmable freezer. The freezing program consisted of the following rates: −4°C/min from 4°C to 0°C, −1°C/min from 0°C to −4°C, −12°C/min from −4°C to −40°C, −30°C/min from −40°C to −140°C. The straws were finally plunged into liquid N_2_ (at −196°C) for further storage. For the analysis, five paillettes were stored for each ejaculate at the Department of BCA and the order in which samples were analyzed in the different tests was determined randomly. For each sample, two straws were used as technical replicates. Each straw was thawed in water at 37°C for 30 s, washed with two centrifuge cycles for 800g x 3 min in 1 mL of Tyrode's medium, and resuspended in the same medium to obtain a final concentration of 25 x 10^6^ sperm/mL.

### 2.3 Motility

Sperm samples were assessed for motility by a computer-assisted sperm analysis system (CASA, Sperm Class Analyzer^®^, Microptic, Barcelona, Spain) before and after the freezing–thawing protocol, using the standard bull setup (60 frames per second; min contrast, 35 min cell size, 8 pixels; progressive cells, VAP ≥ 25.0 μm/s; straightness, ≥ 75%; motile cell cutoff, VAP ≥ 25 μm/s, VSL ≥20.0 μm/s). CASA assessment was validated using previously collected buffalo semen samples diluted with the same extender to establish accurate size settings, ensuring that only sperm cells were counted and preventing both overestimation and underestimation. Approximately one thousand sperm cells for each sample diluted at 40 x 10^6^ sperm/mL were captured and evaluated using a fixed-height Leja Chamber SC 20-01-04-B (Leja, The Netherlands). Sperm motility parameters were assessed both on fresh (F) samples and after thawing at 0 h (T0) and 3 h of incubation at 37°C (T3). The ejaculates used for the experiment had an average TM of 92% and an average PM of 67%. The following parameters were evaluated: total motility (TM, %), progressive motility (PM, %), curvilinear velocity (VCL, μm/s), average path velocity (VAP, μm/s), straight-line velocity (VSL, μm/s), straightness (STR, %), linearity (LIN, %), average lateral head displacement (ALH, μm), wobble (WOB, %) and beat cross frequency (BCF, Hz). Total motility was defined as the percentage of spermatozoa that showed an average path velocity greater than 25 μm/s and a straight path velocity higher than 20 μm/s, whereas progressive motility was defined as the percentage of spermatozoa that showed an average path velocity greater than 25 μm/s and a straightness greater than 75%.

### 2.4 Flow cytometry assay

The samples were analyzed by CyFlow^®^ Space cytometer and FloMax^®^ operating software (Sysmex-Partec, Gorlitz, Germany) equipped with blue laser (488 nm), red laser (638 nm) and UV laser (375/405 nm). Flow-cytometric analyses were performed on fresh and thawed semen immediately (0 h) and after 3 h of incubation at 37°C. Sperm samples were diluted using Tyrode's modified medium at a final concentration of 1 x 10^6^/mL.

In each tube were added sperm samples diluted in 500 mL of Tyrode's medium and stained with 1 μL Hoechst (DAPI and Hoechst Nucleic Acid Stains, Invitrogen™, ThermoFisher) with a final concentration of 200 nM. This dye, known for its ability to bind to DNA, emits blue fluorescence when excited by ultraviolet light and detected by FL4 photomultipliers, allowing to visualize and stain DNA in various biological samples. For all the tubes, debris (non-sperm events) were gated out on the basis of forward scatter (FSC) and side scatter (SSC) dot plot by drawing a region enclosing the cell population of interest; additionally, only the cells positive for Hoechst staining were considered in this region. A total amount of 10,000 cells gated in were recorded.

Acrosomal integrity, intracytoplasmic superoxide and peroxide production, mitochondrial integrity and superoxide production and mitochondrial membrane potential were assessed with the following dyes combination (see Table 1).

**Table 1:** Flow cytometric dye combination.

		**Fluorescence (FL)**			
**Sperm cell parameters**	**UV**	**FL1**	**FL2**	**FL3**	**FL5**
Acrosome integrity	Hoechst	PSA		PI	
Intracellular ROS concentrations	Hoechst	CellROX		PI	
Intracellular peroxide production	Hoechst	CM-H_2_DCFDA		PI	
Intracellular superoxide production	Hoechst	SYBER-14		DHE	
Mitochondrial membrane potential analysis	Hoechst	JC-1 (low membrane potential)	JC-1 (low membrane potential)		
Mitochondrial integrity and ${\text{O}}_{2}^{•-}$ production	Hoechst	SYBER-14		MitoSOX	MitoTracker

#### 2.4.1 Acrosome integrity

Acrosomal status were analyzed using 2.5 μL PSA FITC conjugate (PSA; Lectin from *Pisum sativum* (pea), L0770; Sigma-Aldrich^®^, Milan, Italy; in DMSO, 2 μg/mL final concentration, with absorption/emission maxima of 495/525) coupled with 2.5 μL PI (Propidium Iodide −2.4 mM Solution in Water, Invitrogen^TM^, Thermo Fisher Scientific, Waltham, MA, USA; in DMSO, 2.4μM final concentration). This set of probes has been used to assess acrosomal reacted sperm. Acrosome reaction (AR) pattern is detected when it emits green fluorescence detected by FL1 photomultiplier. This staining was coupled with PI that stains spermatozoa with disrupted plasmalemma. Dead spermatozoa therefore emits red fluorescence with absorption/emission maxima of 535/617 nm detected by the FL3 photomultiplier. By this coupling, it was possible to identify four sperm subpopulations: 1) live spermatozoa with intact outer acrosome membrane; 2) live spermatozoa with damaged outer acrosome membrane; 3) dead spermatozoa with intact outer acrosome membrane; 4) dead spermatozoa with damaged outer acrosome membrane. From these populations, the percentage of spermatozoa with damaged acrosomes (LIVE PSA pos) was calculated, considering only the live cell population as 100%.

#### 2.4.2 Intracellular ROS concentrations: total ROS, H_2_O_2_ and ${\text{O}}_{2}^{•}-$

Intracellular ROS amount was detected by CellROX (CellROX™ Green Reagent, Invitrogen™, Thermo Fisher Scientific, Waltham, MA, USA) that emits green fluorescence detected by FL1 photomultiplier. The cell-permeant dye is weakly fluorescent while in a reduced state and exhibits bright green photostable fluorescence upon oxidation by reactive oxygen species (ROS) and subsequent binding to DNA, with absorption/emission maxima of ~485/520 nm. This staining was coupled with PI that stains spermatozoa with disrupted plasmalemma (dead spermatozoa) emitting red fluorescence detected by the FL3 photomultiplier (2.5 μL CellROX in DMSO, 5 μM final concentration, 2.5 μL PI in DMSO, 2.4μM final concentration). Four following populations were identified: 1) live spermatozoa with ROS production; 2) live spermatozoa without ROS production; 3) dead spermatozoa with ROS production; 4) dead spermatozoa without ROS production. From these populations, the percentage of spermatozoa with ROS production (LIVE CR pos) was calculated, considering only the live cell population as 100%.

Intracellular peroxide production was assessed by CM-H_2_DCFDA (CM-H_2_DCFDA (General Oxidative Stress Indicator), Invitrogen™, Thermo Fisher Scientific, Waltham, MA, USA), a chloromethyl derivative of H_2_DCFDA, useful as an indicator for reactive oxygen species (ROS) in cells, and emits green fluorescence upon oxidation by H_2_O_2_, excited at ~492–495 nm and emits in 517–527 nm. This stain passively diffuses into cells, where its acetate groups are cleaved by intracellular esterases and its thiol-reactive chloromethyl group reacts with intracellular glutathione and other thiols. It was coupled with PI that stains spermatozoa with disrupted plasmalemma (dead spermatozoa) emitting red fluorescence detected by the FL3 photomultiplier (2.5 μL CM-H_2_DCFDA in DMSO, 5 μM final concentration, 2.5 μL PI in DMSO, 2.4μM final concentration). Samples were incubated at 37°C for 20 min in the dark. The H_2_O_2_ production was assessed by H_2_CDFDA oxidation in viable cells. Four following populations were identified: 1) live spermatozoa with H_2_O_2_ production; 2) live spermatozoa without H_2_O_2_ production; 3) dead spermatozoa with H_2_O_2_ production; 4) dead spermatozoa without H_2_O_2_ production. From these populations, the percentage of spermatozoa with peroxide production (LIVE CM-H2DCFDA pos) was calculated, considering only the live cell population as 100%.

Intracellular superoxide production was detected by dihydroethidium (DHE; Dihydroethidium (Hydroethidine), Invitrogen™, Thermo Fisher Scientific, Waltham, MA, USA), a cell-permeable dye emitting blue-fluorescence in the cytosol until oxidation mainly by superoxide radicals (${\text{O}}_{2}^{•-}$), where it intercalates within the cell's DNA, staining its nucleus a bright fluorescent red (610 nm), with absorption/emission maxima of 518/606 nm; the staining was coupled with SYBR-1414 (SYBR^®^ Green I nucleic acid gel stain, 10,000 × in DMSO, Sigma-Aldrich^®^, Milan, Italy), that stains the head of viable spermatozoa in green, excited at 480 nm and emits in 520 nm. Sample were stained with 2.5 μL DHE in DMSO (5 uM final concentration), 2.5 μL SYBR-14 in DMSO (0.005x) and were incubated at 37°C for 20 min in the dark and ${\text{O}}_{2}^{•-}$ production was assessed on non-apoptotic cells. Four following populations were identified: 1) live spermatozoa with ${\text{O}}_{2}^{•-}$ production; 2) live spermatozoa without ${\text{O}}_{2}^{•-}$ production; 3) dead spermatozoa with ${\text{O}}_{2}^{•-}$ production; 4) dead spermatozoa without ${\text{O}}_{2}^{•-}$ production. From these populations, the percentage of live spermatozoa with ${\text{O}}_{2}^{•-}$ production (LIVE DHE pos) was calculated, considering only the live cell population as 100%.

#### 2.4.3 Mitochondrial membrane potential analysis

5,5′,6,6′-tetrachloro-1,1′,3,3′-tetraethylbenzimidazolyl carbocyanine iodide (JC-1, synonyms: CBIC2, MedChemExpress, Monmouth Junction, NJ, USA) was used to detect mitochondrial membrane potential. JC-1 accumulates in mitochondria in a potential dependent manner and can be used to detect the membrane potential of cells, tissues or purified mitochondria. In normal mitochondria with high membrane potential, JC-1 forms multimers (known as JC1-aggregates) and emits red fluorescence (Ex = 488 nm, Em = 595 nm), which is detected by FL-2 photomultiplier. In contrast, when mitochondria have low membrane potential, JC-1 maintains its monomeric form (M-band) in the matrix of mitochondria and emits green fluorescence (Ex = 488 nm, Em = 530 nm), which is detected by FL-1 photomultiplier. Samples were stained with 2.5 μL JC1 (at a final concentration of 1 μM) and successively incubated at 37 °C for 20 min in the dark. Cells were analyzed in a FL-1/FL-2 plot. High mitochondrial membrane potential cells (HMMP) stained orange (higher FL-2) and low mitochondrial membrane potential cells (LMMP) stained green (higher FL-1).

#### 2.4.4 Mitochondrial integrity and ${\text{O}}_{2}^{•}-$ production

MitoSOX Red (MitoSOX™ Mitochondrial Superoxide Indicators, for live-cell imaging, Invitrogen™, Thermo Fisher Scientific, Waltham, MA, USA) is a lipid-soluble, cell-permeable cation that selectively targets the mitochondrial matrix and can detect superoxide radicals (${\text{O}}_{2}^{•-}$) generation in this organelle. MitoSOX Red (MX) emits red fluorescence upon oxidation, with absorption/emission maxima of ~396/610 nm. It was coupled with SYBR-14 as a counterstaining to distinguish sperm with early changes in membrane permeability subpopulation from the intact ones. It was coupled with SYBR-14 as a counterstaining to distinguish sperm with early changes in membrane permeability subpopulation from the intact ones. Stains sperm with early changes in membrane permeability and emit green fluorescence upon binding to DNA, detected by the FL1 photomultiplier. The MitoTracker deep red probe (MT; MitoTracker™ Dyes for Mitochondria Labeling, Invitrogen™, Thermo Fisher Scientific, Waltham, MA, USA) was included to assess mitochondrial integrity simultaneously. MT is excited by the red diode laser, with absorption/emission maxima of 644/665 nm.

Sperm samples were stained with 2.5 μL SYBR-14 (in DMSO, 0.005 x), 1 μL MX (in DMSO, 1 μM final concentration), and 2.5 μL MT (in DMSO, 100 nM final concentration). Samples were incubated at 37°C for 20 min in the dark.

The mitochondrial production of ${\text{O}}_{2}^{•-}$ by live cells with intact mitochondria was recorded in this analysis. In this study, we used the population of live spermatozoa, distinguishing cells with high and low ${\text{O}}_{2}^{•-}$ generation in the subpopulations with intact mitochondria or damaged mitochondria. The live population with mitochondrial superoxide production (considering both cells with intact and damaged mitochondria) was named LIVE MX pos. The live population with mitochondrial integrity (considering both cells positive and negative to MX) was named LIVE MT pos.

#### 2.4.5 Sperm membrane integrity

Sperm viability (LIVE) was calculated by determining the mean percentage of non-viable cells (PI-positive) as measured by multiple assays, previously described, that used propidium iodide (PI) as a counterstain (Table 1).

### 2.5 Statistical analysis

Statistical analyses were performed using R (version 4.3.0). The correlation between motility and flow cytometry parameters were determined by Spearman test for non-parametric variables. In addition, linear mixed models were utilized to investigate the relation between the motility or flow cytometry parameters and semen storage conditions. For each model, motility or flow cytometry parameters were used as dependent variables, storage time (3 levels: F, T0, T3) as fixed factor and bull as random factor. The residues were checked for variance homogeneity and normal distribution (30). To study the relationship between sperm motility and flow cytometric parameters, thawed samples were clustered based on TM and PM measured at T0 by the k-means methods, resulting in a high motility (HM; *N* = 10) group and a low motility (LM; *N* = 7) groups. Differences between groups were studied using the mixed linear models option (of R).

For each model the flow cytometry parameters were the dependent variables, time after thawing (2 levels: T0; T3) and the motility groups (2 levels: HM and LM) were the fixed factors, while bull was used as random factor. For each model, the residues were checked for variance homogeneity and normal distribution (30). Values are expressed as mean ± standard deviation (SD), unless otherwise specified, and level of significance was set at *P* < 0.05.

## 3 Results

### 3.1 Correlations

As shown in Table 2, all motility parameters significantly strongly correlated each other's (data not shown). TM and PM showed strong significant positive correlations (*p* < 0.05) with mitochondrial integrity (LIVE MT pos; TM: *r* = 0.55; PM: *r* = 0.59) and high mitochondrial membrane potential (HMMP; TM: *r* = 0.75; PM: *r* = 0.77). In addition, TM and PM were moderately positively correlated (*p* < 0.05) with LIVE CR pos (TM: *r* = 0.52; PM: *r* = 0.50) and weakly correlated with LIVE CM-H_2_DCFDA pos (TM: *r* = 0.32; PM: *r* = 0.46). Live spermatozoa that produce mitochondrial superoxide (LIVE DHE pos) were negatively correlated with TM (*r* = −0.52; *p* < 0.05), PM (*r* = −0.74; *p* < 0.05), HMMP (*r* = −0.46; *p* < 0.05), LIVE MT pos (*r* = −0.58; *p* < 0.05) and LIVE CM- H_2_DCFDA pos (*r* = −0.58; *p* < 0.05). On the contrary the LIVE CR pos and LIVE CM-H_2_DCFDA pos were positively correlated with mitochondrial activity and integrity (Table 2) and negatively correlated with LIVE DHE pos (*r* = −0.48; *p* < 0.05). The acrosome integrity of live sperm is not included in the table as it did not show significant correlations with any of the parameters analyzed.

**Table 2:** Correlation between different parameters of semen quality.

**Semen parameters**	**TM**	**PM**	**LIVE CR pos**	**LIVE CM-H_2_DCFDA pos**	**LIVE DHE pos**	**LIVE MT pos**	**LIVE MX pos**	**HMMP**
TM		0.84	0.52	0.32	−0.36	0.55	−0.52	0.75
PM			0.50	0.46		0.59	−0.74	0.77
LIVE CR pos					−0.38	0.50	−0.34	0.49
LIVE CM- H_2_DCFDA pos						0.39	−0.58	
LIVE DHE pos						−0.45	0.31	
LIVE MT pos							−0.58	0.64
LIVE MX pos								−0.46
HMMP								

The significant correlations (*p* < 0.05) with a *r*≥ 0.30 are considered. Correlations with a *r*≥ 0.40 are highlighted in orange while the significant correlations (*p* < 0.05) with 0.30 ≤ *r* < 0.40 are highlighted in light orange. Correlations with r ≤ 0.20 are not included.

### 3.2 Motility

The motility parameters analyzed showed a significant decrease between fresh semen and thawed semen. In particular, TM, PM, VAP, VCL, VSL, LIN, BCF, WOB, ALH and STR were significantly lower (*p* < 0.05) in the thawed semen at 3 h compared to the thawed semen at 0 h and the fresh semen (Table 3).

**Table 3:** Motility parameters of fresh (F) and frozen-thawed semen at 0 h (T0) and 3 h (T3) incubation time at 37°C.

**Motility parameters**	**F**	**T0**	**T3**	**R^2^ model**
TM	92.1 ± 5.66^a^	72.61 ± 18.62^b^	47.34 ± 22.62^c^	0.58
PM	67.56 ± 7.42^a^	39.81 ± 14.09^b^	12.34 ± 13.29^c^	0.79
VAP	99.47 ± 22.93^a^	37.76 ± 15.52^b^	17.05 ± 11.87^c^	0.84
VSL	67.79 ± 21.44^a^	28.43 ± 13.35^b^	8.14 ± 10.68^c^	0.89
VCL	120.29 ± 32.48^a^	61.48 ± 21.93^b^	33.61 ± 19.26^c^	0.72
STR	75.85 ± 9.49^a^	61.60 ± 7.32^b^	39.64 ± 11.14^c^	0.70
LIN	55.29 ± 8.06^a^	36.03 ± 8.63^b^	17.35 ± 8.09^c^	0.75
ALH	1.74 ± 0.18^a^	1.34 ± 0.31^b^	0.96 ± 0.33^c^	0.54
BCF	24.42 ± 10.61^a^	10.48 ± 4.62^b^	3.88 ± 3.94^c^	0.67
WOB	69.85 ± 5.16^a^	54.92 ± 7.01^b^	47.03 ± 9.36^c^	0.74

The lower-case letters indicate significantly different means (*p* < 0.05) for each variable between groups (F vs T0 vs T3).

### 3.3 Flow cytometry assay

In Table 4 the differences of flow-cytometry parameters are shown between fresh and thawed semen at 0 h and at 3 h post incubation. A significant decrease in sperm viability was observed in thawed semen compared to fresh semen. For most assessments the percentages of cell populations are expressed relative to live spermatozoa, while the percentage of HMMP cells is expressed relative to the total population of spermatozoa. A significant reduction in the percentage of LIVE CR pos cells (T0: 83.22 ± 14.14; T3: 58.71 ± 21.21; *p* = 0.0002) and LIVE MT pos (T0: 88.8 ± 12.37; T3: 63.77 ± 22.18; *p* < 0.0001) was observed at 3 h post thawing. Meanwhile the cytoplasmic and mitochondrial superoxide production increased in live spermatozoa at 3 h post incubation (LIVE DHE pos; T0: 23.04 ± 18.64; T3: 52.64 ± 23.87, *p* = 0.0002; LIVE MX pos; T0: 14.74 ± 14.43; T3: 43.59 ± 24.49, *p* < 0.0001). Moreover, after thawing it was observed an increase of the percentage of LIVE CM-H_2_DCFDA pos (F: 23.02 ± 7.42; T0: 11.49 ± 8.85; *p* < 0.0001) and a significantly decrease of the percentage of HMMP (F: 83.29 ± 11.20; T0: 56.98 ± 15.87; *p* < 0.0001). No significant differences were observed in the other flow cytometric parameters analyzed.

**Table 4:** Flow-cytometry parameters of fresh (F) and frozen-thawed semen at 0 h (T0) and 3 h (T3) storage time.

**Flow-cytometry parameters**	**F**	**T0**	**T3**	**R^2^ model**
LIVE	59.58 ± 11.5^a^	43.78 ± 14.24^b^	34.68 ± 17.33^b^	0.68
LIVE PSA pos	21.46 ± 19.94^a^	29.9 ± 17.62^a^	19.19 ± 15.94^a^	0.06
LIVE CR pos	85.01 ± 18.7^a^	83.22 ± 14.14^a^	58.71 ± 21.21^b^	0.31
LIVE DHE pos	35.43 ± 22.03^a^	23.04 ± 18.64^a^	52.64 ± 23.87^b^	0.48
LIVE CM-H_2_DCFDA pos	23.02 ± 7.42^a^	11.49 ± 8.85^b^	9.38 ± 7.11^b^	0.42
LIVE MX pos	4.56 ± 3.64^a^	14.74 ± 14.43^a^	43.59 ± 24.49^b^	0.53
LIVE MT pos	96.33 ± 1.72^a^	88.8 ± 12.37^a^	63.77 ± 22.18^b^	0.57
HMMP	83.29 ± 11.20^a^	56.98 ± 15.87^b^	44.64 ± 15.23^b^	0.60

Lower-case letters (^a, b^) in the rows indicate significantly different means (p < 0.05).

### 3.4 Relationship between motility groups and flow-cytometry parameters

**Figure 1:**
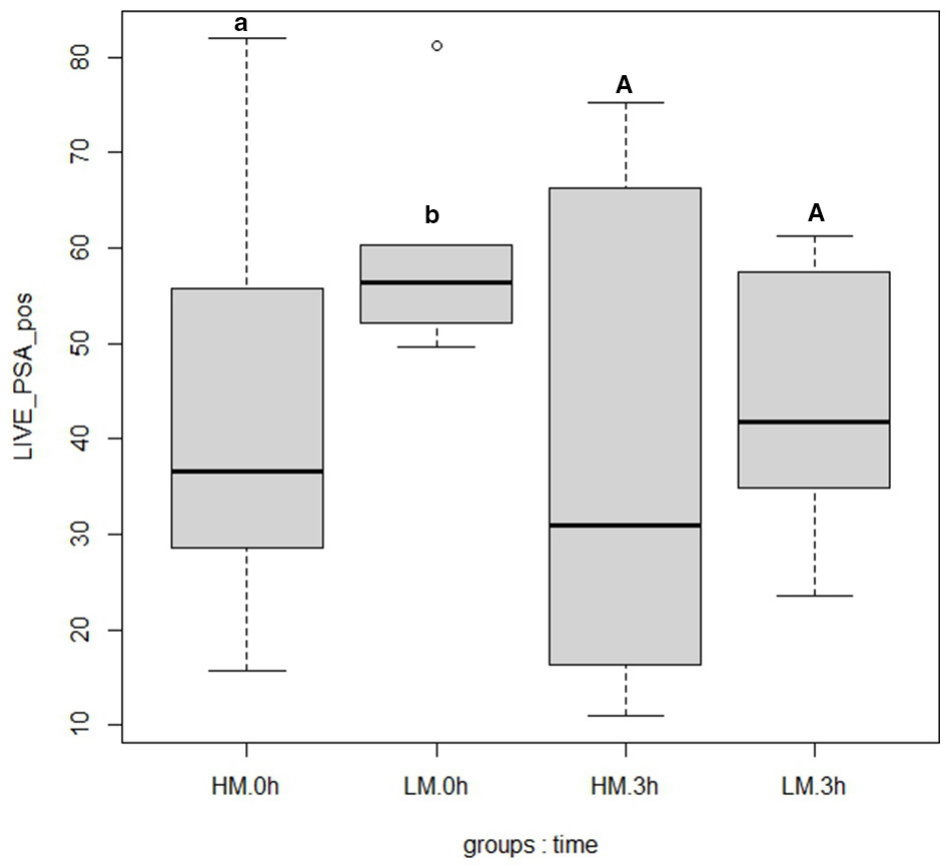
Box plot of acrosome integrity between high motility (HM; *N* = 10) and low motility (LM; *N* = 7) groups at 0 h (T0) and 3 h (T3) after thawing. Differences between groups were studied using the mixed linear models option (of R). Different letters indicate significantly different means (*p* < 0.05) between groups (HM and LM) within the same post-thawing time point (0 h and 3 h).

For the two groups LM and HM, no significant variation in most of the flow-cytometry parameters were observed (Table 5). The only significant difference observed (*p* < 0.05) is the acrosome integrity of live spermatozoa. As shown in Figure 1, the LM group exhibited greater acrosomal damage in thawed semen at 0 h (44.94 ± 7.36) compared to the HM group (19.16 ± 14.49).

**Table 5:** Flow-cytometry parameters of HM group and LM group at 0 h (T0) and 3 h (T3) storage time.

**Flow-cytometry parameters**	**T0**		**T3**		**R^2^ model**
	**LM**	**HM**	**LM**	**HM**	
LIVE	40.01 ± 16.84^a*^	47.54 ± 11.07^a*^	25.14 ± 23.28^A^	39.45 ± 12.71^A^	0.73
LIVE PSA pos	44.94 ± 7.36^a*^	19.16 ± 14.49^b^	24.10 ± 19.27^A^	16.13 ± 13.97^A^	0.23
LIVE CR pos	81.21 ± 17.89^a*^	84.63 ± 11.71^a*^	55.37 ± 28.54^A^	60.71 ± 16.89^A^	0.49
LIVE DHE pos	19.75 ± 11.69^a*^	25.34 ± 22.67^a*^	53.97 ± 21.90^A^	51.71 ± 26.28^A^	0.51
LIVE CM-H_2_DCFDA pos	12.46 ± 12.66^a^	10.82 ± 5.58^a^	9.32 ± 5.08^A^	9.42 ± 8.53^A^	0.42
LIVE MX pos	12.42 ± 15.40^a*^	17.06 ± 14.19^a*^	42.27 ± 26.74^A^	44.51 ± 24.23^A^	0.53
LIVE MT pos	94.24 ± 15.69^a*^	83.37 ± 4.02^a*^	68.03 ± 6.30^A^	60.79 ± 28.70^A^	0.64
HMMP	50.50 ± 17.02^a^	61.52 ± 14.11^a^	45.61 ± 13.92 ^A^	44.00 ± 16.84^A^	0.63

Different lowercase and uppercase letters (^a, A^) in the rows indicate significantly different means (p < 0.05) within the same incubation time (0 h and 3 h, respectively). The asterixis indicate differences between storage time within the same motility group.

## 4 Discussion and conclusion

### 4.1 Physiological parameters and mitochondrial function

Despite the existence of numerous studies evaluating semen quality in various river buffalo breeds, to the best of our knowledge, this is the first study on IMB to investigate the relationship between intracellular oxidative status parameters and mitochondrial functionality and the effects of the cryopreservation procedure on these parameters. Our analyses showed that the motility in fresh semen was considerably higher than what reported in previous research on IMB (92.1 ± 5.66%), and that it dramatically decreased at 0 and 3 h after incubation. As reported in previous studies comparing motility assessments across various CASA systems, setting used and operator conditions is challenging (31). Additionally, our findings support the notion that cryopreservation in IMB semen presents similar difficulties as observed in other mammalian species and buffalo breeds (16, 17). Lower TM percentage were observed after cryopreservation in the other studies [average 40% in **(author?)** (18); 52% in **(author?)** (3)]. The reduced motility caused by freezing-thawing protocol is widely studied and is due to various factors, including ice crystal formation, osmotic stress, membrane damage, mitochondrial dysfunction, oxidative stress, and cytoskeletal alterations (32). Sperm viability significantly decreased after cryopreservation and remained unchanged after 3 h of incubation at 37°C. Therefore, while 3 h of incubation significantly affected motility did not influence semen viability, resulting in an increased static viable spermatozoa.

The acrosome integrity is essential at the time of fertilization (33). In most buffalo breeds, more than 80% of sperm in fresh semen were found to have intact acrosome, regardless of the method used for the assessment (34–36). In our study, the acrosome integrity percentage of live spermatozoa was similar to what found in the other studies on IMB breed (3, 7). Several studies demonstrated that cryopreservation significantly affects acrosome integrity of bull and boar semen (37–39). Results of the present study showed that, unlike other species, cryopreservation and incubation cause a lower acrosomal damage in IMB spermatozoa. A possible explanation could be related to differences in membrane composition and acrosomal cryotolerance, a phenomenon already described in other mammalian species (40). Whether similar mechanisms occur in buffalo remains to be clarified and warrants further investigation.

Our results showed that the percentage of spermatozoa with high mitochondrial membrane potential (HMMP) significantly decreased after cryopreservation, while the membrane mitochondrial integrity is significantly reduced (*p* < 0.05) only after 3 h of incubation post thawing. The significant positive correlations between motility (TM and PM) and HMMP suggest a fundamental role of mitochondria in buffalo sperm energy metabolism. Mitochondria are the central hub for sperm metabolism, and control numerous functions in spermatozoa, including the regulation of their lifespan, Ca^2+^ homeostasis and signaling functions (41). Energy metabolism consists of a series of reactions in which biological molecules are oxidized to simpler ones, and the energy released is used to phosphorylate adenosine diphosphate (ADP) to adenosine triphosphate [ATP; (42, 43)]. Our results suggest that the reduction in motility of cryopreserved semen is associated with decreased mitochondrial activity, which is negatively correlated with mitochondrial superoxide production. As confirmed by several studies, mitochondrial superoxide production is related to the uncoupling of mitochondrial complexes and is evidence of mitochondrial damage (29, 44–46).

The observed positive relationship between mitochondrial functionality and semen motility support the hypothesis that sperm cells heavily depend on mitochondrial activity for their energy production and movement. Indeed, other studies have shown that depending on the species, spermatozoa utilize different metabolic pathways to sustain the motility, even after the cryopreservation process. As regards the boar, it has been shown that sperm use mainly anaerobic metabolism (8, 47), although active metabolism of mitochondrial substrates has been demonstrated (48). Horse sperm are mostly oxidative cells that require intact mitochondria for their functionality and survival (49–52), while bull spermatozoa can rely both on glycolysis and oxidative phosphorylation pathways (8, 29). Whereas the metabolic pathways of sperm from stallions, bulls, and boars have been extensively studied, the preferred metabolic pathway for energy production in buffalo spermatozoa are currently unknown. Our preliminary findings suggest that mitochondrial function is pivotal in maintaining sperm motility and is compromised by the process of semen cryopreservation. Further studies should investigate the impact of mitochondrial complex inhibitors and different storage conditions (aerobic vs. anaerobic) on sperm energy metabolism.

### 4.2 Intracellular ROS production

Another key point to consider is the different trends of ROS production after cryopreservation and their different correlation with semen parameters. Cryopreservation is one of the processes that leads to the increase in ROS production with a reduction of viability, motility, cellular enzymatic activity, and fertilizing ability of sperm in different mammal species (23, 29, 53–55). In our study, live spermatozoa from fresh semen samples exhibited a high level of total ROS production, as detected by the CellROX™ Green Reagent, with an average of 85% of live cells being positive. The live cells positive to CR showed a positive correlation with mitochondrial integrity and activity, suggesting that elevated ROS levels may reflect enhanced mitochondrial activity. According to Thermo Fisher Scientific (56) and the recent literature (57), CellROX™ Green is sensitive to a broad spectrum of oxidative conditions, reacting with various ROS, including superoxide anion (${\text{O}}_{2}^{-}\cdot$), hydroxyl radical (·OH), peroxynitrite (ONOO^−^), and hydrogen peroxide (H_2_O_2_). A higher ROS production was observed not only in cryopreserved semen but also in fresh samples. Additionally, the 3-h incubation post thawing resulted in a decrease in cytoplasmic ROS levels, contrary to our initial hypothesis. The elevated levels of ROS observed in buffalo semen may be associated with higher metabolic activity, and do not inherently imply the presence of oxidative cellular damage. This increased metabolic rate, however, necessitates a robust antioxidant system to effectively counterbalance the heightened ROS production. These observations support the “paradoxical effect” of oxidative stress on spermatozoa physiology described by Gibb et al. (52), who found positive correlations between sperm motility, ROS production and lipid peroxidation. Similarly, Vigolo et al. (58) reported significant positive correlations between advanced oxidative protein products (AOPP) in seminal plasma and the motility and viability of both fresh and cryopreserved bull semen. These findings suggest that seminal plasma proteins may play a protective role as scavengers against increased intracellular ROS production. Key enzymes involved in sperm antioxidant systems include glutathione, peroxiredoxins, thioredoxin, MnSOD, which help prevent irreversible damage on sperm DNA, membranes and mitochondria (50). Further investigations should evaluate the antioxidants activity on fresh and cryopreserved semen as well as structural cell damage and the activities of antioxidant enzymes. Indeed, only a few studies which involved a limited number of IBM subjects, such as Minervini et al. (7), have investigated DNA integrity by different flow cytometry assays in relation to fertility. The study found a high variability between each buffalo bull but generally good DNA integrity. Moreover, the study of Serafini et al. (3) provided reference values of sperm DNA integrity using three different assays. Other evaluations of nuclear and membrane damage should be conducted in relation to the intracellular REDOX status.

Interestingly, ROS production measured by CellROX™ Green showed a weak negative correlation with both intracytoplasmic superoxide (DHE-positive cells) and mitochondrial superoxide (MitoSOX™-positive cells), which positive correlate each other's. This finding suggests a different biological meaning of the different ROS species detected by these probes and compartmental differences in ROS dynamics. On the one hand, both H_2_O_2_ and total ROS levels significantly decreased (*p* < 0.01) in thawed semen, either immediately after thawing or following a 3 h incubation period. This reduction suggests a decline in energetic metabolism. On the other hand, both mitochondrial and intracytoplasmic superoxide production significantly increased (*p* < 0.01) after 3 h of incubation, suggesting mitochondrial leakage and consequent cellular damage. Indeed, intracytoplasmic superoxide production negatively correlated with HMMP, as previously mentioned. Additionally, DHE-positive cells showed a moderate negative correlation with mitochondrial integrity. These findings allow us to speculate that the intracytoplasmic superoxide detected by DHE could partly reflect increased mitochondrial superoxide production after 3 h of incubation, although this mechanism cannot be confirmed with the present data. While the diffusion of ${\text{O}}_{2}^{•-}$ across membranes is typically limited due to its anionic nature, the observed disruption of mitochondrial membranes after 3 h of incubation could facilitate increased ${\text{O}}_{2}^{•-}$ diffusion in the cytoplasm. Superoxide Dismutase (SOD) is the primary enzyme responsible for converting superoxide (${\text{O}}_{2}^{•-}$) into hydrogen peroxide (H_2_O_2_) (71). Subsequently, spermatozoa employ multiple enzymatic systems to eliminate excess hydrogen peroxide (H_2_O_2_), primarily through glutathione peroxidases (GPXs), catalase, and peroxiredoxins (41, 59). In our study, we observed a decrease in intracellular peroxide production (LIVE DCFDA pos) immediately after thawing, which remained consistently low after 3 h of incubation. This finding contrasts with the increase in cellular superoxide production observed after 3 h of incubation post-thawing. The observed reduction in cytosolic H_2_O_2_ levels in thawed semen at T0 and T3 compared to fresh ejaculate may be attributed to decreased activity of various SOD enzymes, disrupting the balance between superoxide production and its conversion to hydrogen peroxide (59). This hypothesis is further corroborated by the simultaneous increase in MX-positive cells, which, along with DHE-positive cells, exhibited a significant negative correlation with DCFDA-positive cells. As demonstrated by other studies, this decline in SOD activity compared to the fresh semen may be due to the consumption of SOD in neutralizing superoxide radicals, or its partial inactivation during the equilibration and freeze-thaw processes (60, 61).

Previous research on cryopreserved buffalo semen has examined both antioxidant activity and oxidative status during the cryopreservation process using spectroscopic methods. These studies have revealed a significant reduction in superoxide dismutase (SOD) levels in cryopreserved semen. Additionally, they have identified notable variations in SOD levels among individual buffaloes (61, 62). Nevertheless, the complexity of buffalo breeds and species, as well as environmental factors and housing conditions that may influence the results make a direct comparison with these studies difficult. One of the few studies on IMB buffalo by Topraggaleh et al. (63) reported higher motility, viability and mitochondrial activity in frozen-thawed IMB semen imported in Iran, than in the native Iranian Azari buffaloes. Beyond the different temperature, humidity and day length conditions, semen processing, including dilution, type of extender, equilibration, freezing, and thawing, play a significant role in determining the post-thaw quality of buffalo spermatozoa. The complex interplay of these factors underscores the importance of optimizing each step in the cryopreservation process to maintain sperm viability and functionality.

In our study, a 3 h incubation post-thawing generally resulted in significant changes in intracellular ROS levels and a decrease in mitochondrial integrity. This phenomenon was not unique to IMB, as similar observations were made following the incubation of thawed semen from other species as well (54, 64, 65). The thawed semen was incubated for 3 h at 37°C to mimic its lifespan and resistance in the female oviductal tract but this type of incubation is completely different from the oviductal environment, and it does not consider presence and effect of the oviductal proteins. In Murrah buffalo, oviductal fluid provides an optimal environment for sustaining sperm functions until the oocyte arrives after ovulation (66). This protective effect might be due to certain proteins/factors in the oviduct during estrus (non-luteal phase) that protect sperm from oxidative and proteolytic damage and maintain its membrane integrity (67). Functional studies indicate that oviductal specific glycoproteins secreted from oviductal epithelium have a direct effect on maintaining motility, viability, acrosomal integrity (25, 67, 68) and induce sperm capacitation (69). Unlike with other breeds, could be indicative of its higher metabolic activity in the Italian Mediterranean Buffalo (IMB) is limited. The oviductal/uterine environment can significantly influence the functional parameters of semen, and its effects could be indirectly assessed through conception rates.

In the last part of our experiment, thawed semen samples of IMB bulls were divided into two different groups according to motility parameters allowing us to obtain one group with higher motility individuals (HM) and another with low motility ones (LM). Motility parameters were able to predict the *in vivo* fertility of buffalo bull (33) and poor sperm motility has been correlated with low fertility in buffalo (70). In our study the only parameter that was significantly different between the two groups was the percentage of live spermatozoa with intact acrosome (LIVE PSA spermatozoa), which was significantly lower in HM group. In addition, HMMP showed a much greater variability in distribution in the low motility (LM) group compared to the high motility (HM) group (Table 5). Viability showed no significant difference between the high motility (HM) and low motility (LM) groups; therefore, we assume that the LM group has a much higher proportion of live but static spermatozoa than the HM group. The limited sample size of this study may have contributed to the lack of significant differences in physiological parameters of cryopreserved semen that could predict higher or lower motility groups. Although oxidative stress parameters showed significant variations before and after cryopreservation, they did not differ between the two motility groups in cryopreserved semen. These findings underscore the need for more comprehensive analyses, including evaluation of intracellular antioxidant content and cellular damage associated with cryopreservation. Future research should focus on investigating the impact of cryopreservation on oxidative balance and its subsequent effects on sperm motility in preserved semen samples.

To our knowledge, this is the first study that thoroughly investigated the relationship between mitochondrial status and sperm functionality before and after cryopreservation in IMB semen.

Our results indicate, in accordance with our hypothesis, a pivotal role of mitochondria in sustaining motility and confirmed the deleterious effects of cryopreservation on motility parameters and mitochondrial activity in IMB, although further research is needed to explore the balance between aerobic and anaerobic pathways. This study reveals how different ROS species exhibit varied relationships with semen characteristics. Notably, we observed a negative impact of intracytoplasmic and mitochondrial superoxide production on semen parameters, which showed an opposite trend compared to other ROS species. Contrary to our initial hypothesis of a general increase in intracellular ROS amount after cryopreservation our results reveal a more nuanced picture, suggesting different biological roles for various ROS species. The main limitation of this study is that we did not assess the antioxidant activity in buffalo semen samples and whether the observed increase of ROS production actually lead to oxidative damages of macromolecules. Thus, further investigation should analyze the antioxidant content and assess cellular damage to determine whether and how the intracellular redox balance is affected by cryopreservation.

A deeper understanding of IMB sperm energy metabolism and its relationship with redox regulation could lead to advancements in current assisted reproductive technologies (ART) because these factors play a crucial role in sperm viability, motility, and fertilization capacity, which are key for optimizing outcomes in ART procedures. This improved knowledge may also offer new insights into the causes of IMB male infertility and the effect of seasonality on specific semen physiological parameters, as variation in sperm energy metabolism and redox balance can fluctuate significantly with seasonal changes, potentially affecting male reproductive performance under different environmental conditions.

## Data Availability

The raw data supporting the conclusions of this article will be made available by the authors, without undue reservation.
