# Supplementary material for: Mitochondrial function and reactive oxygen species dynamics in Italian Mediterranean buffalo semen following cryopreservation and post-thaw incubation
Source: Front Vet Sci. 2025 Dec 18;12:1733446. doi: 10.3389/fvets.2025.1733446 (PMC12756934; doi:10.3389/fvets.2025.1733446)
Supplement: Supplementary file 1 [file Image_1.pdf]

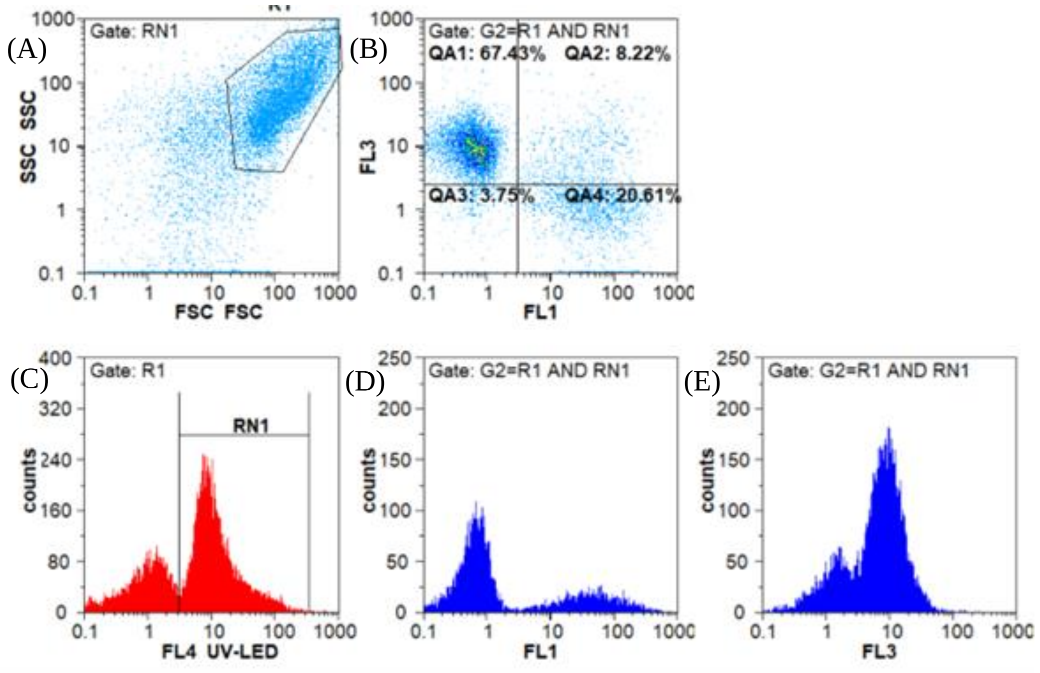

Supplementary Figure 1. Flow cytometry plots showing assay with Hoechst-CR-PI. (A) Density plot showing the combination between SSC and FSC. Cells were gated based on size and granularity using FSC vs SSC (A) and Hoechst positivity (C). (B) Density plot illustrating the combination between FL1 and FL3. (C-D-E) Histograms in FL4, FL1 and FL3.

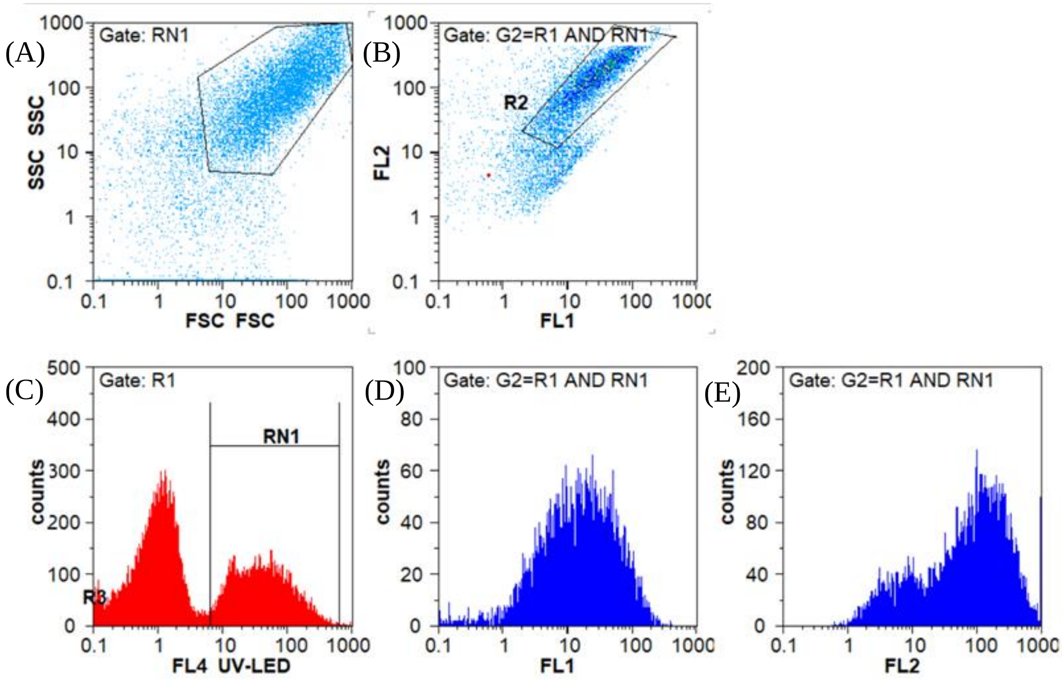

Supplementary Figure 2. Flow cytometry plots showing assay with Hoechst-and JC1. (A) Density plot showing the combination between SSC and FSC. Cells were gated based on size and granularity using FSC vs SSC (A) and Hoechst positivity (C). (B) Density plot illustrating the combination between FL1 and FL2. High mitochondrial membrane potential cells (HMMP) stained orange (higher FL-2) and low mitochondrial membrane potential cells (LMMP) stained green (higher FL-1).

(C-D-E) Histograms in FL4, FL1 and FL2.

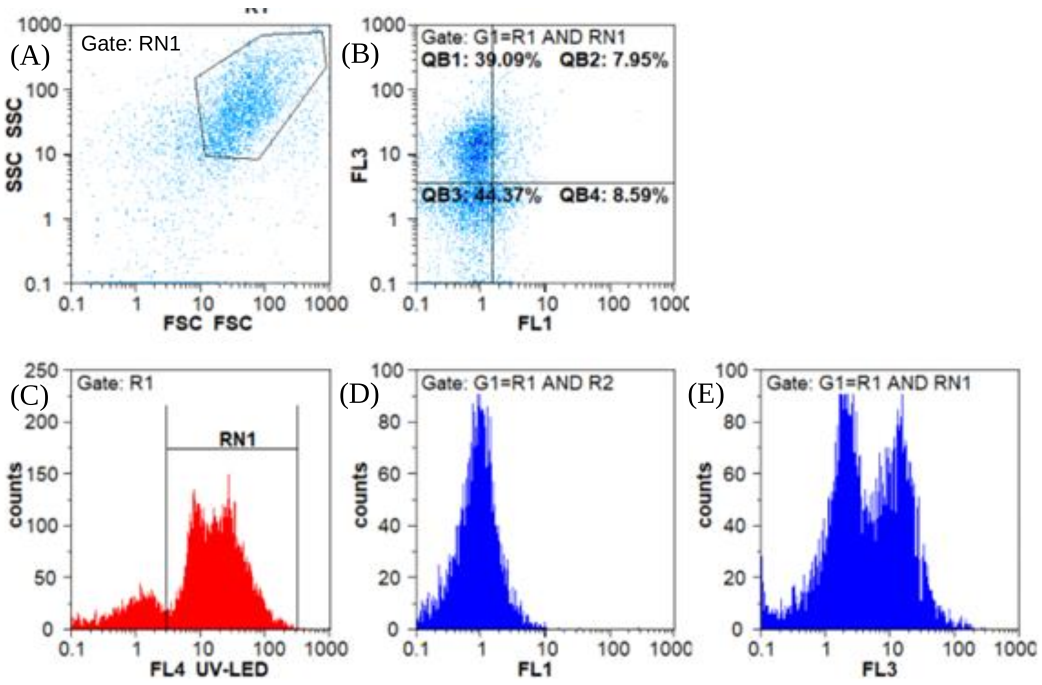

Supplementary Figure 3. Flow cytometry plots showing assay with Hoechst-DCFDA-PI. (A) Density plot showing the combination between SSC and FSC. Cells were gated based on size and granularity using FSC vs SSC (A) and Hoechst positivity (C). (B) Density plot illustrating the combination between FL1 and FL3. (C-D-E) Histograms in FL4, FL1 and FL3.

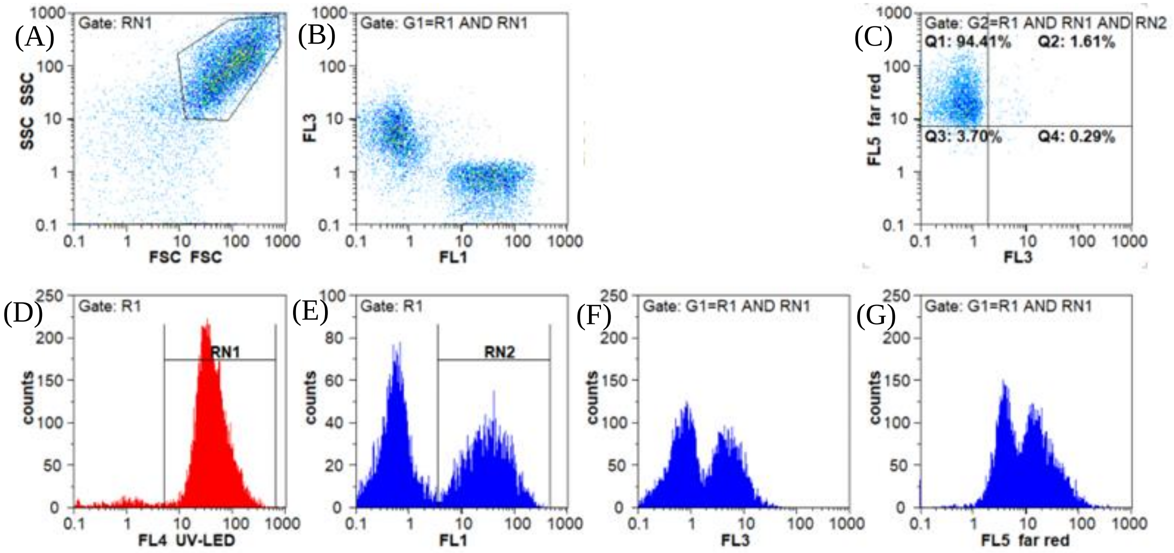

Supplementary Figure 4. Flow cytometry plots showing assay with Hoechst-SG-MX-MT. (A) Density plot showing the combination between SSC and FSC. Cells were gated based on size and granularity using FSC vs SSC (A) and Hoechst positivity (D). (B) Density plot illustrating the combination between FL1 and FL3. (C) Density plot illustrating the combination between FL3 and FL5. In this dot plot were gated only live cells (R1 and RN1 and RN2). (D-E-F-G) Histograms in FL4, FL1, FL3 and FL5.

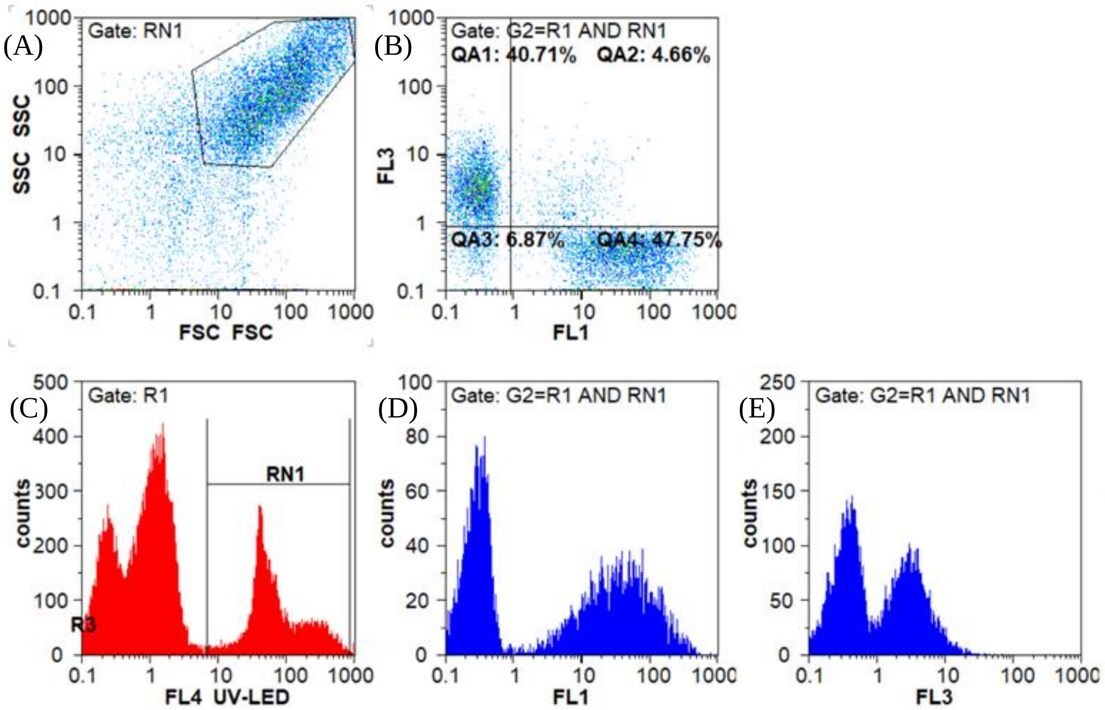

Supplementary Figure 5. Flow cytometry plots showing assay with Hoechst-SG-DHE. (A) Density plot showing the combination between SSC and FSC. Cells were gated based on size and granularity using FSC vs SSC (A) and Hoechst positivity (C). (B) Density plot illustrating the combination between FL1 and FL3. (C-D-E) Histograms in FL4, FL1 and FL3.

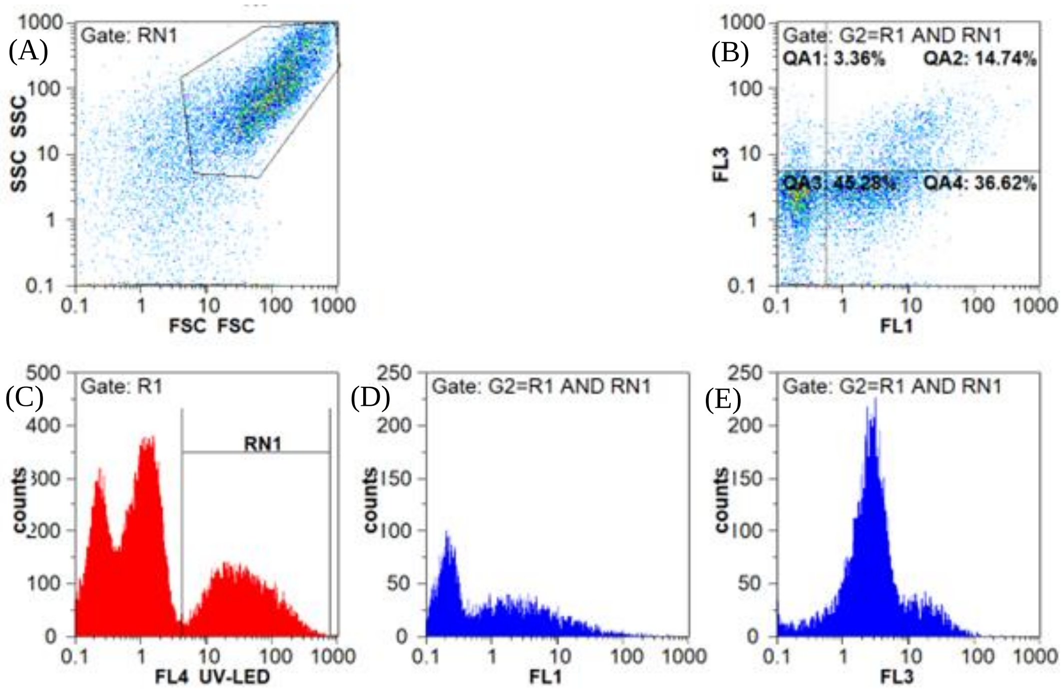

Supplementary Figure 6. Flow cytometry plots showing assay with Hoechst-PSA-PI. (A) Density plot showing the combination between SSC and FSC. Cells were gated based on size and granularity using FSC vs SSC (A) and Hoechst positivity (C). (B) Density plot illustrating the combination between FL1 and FL3. (C-D-E) Histograms in FL4, FL1 and FL3.
